# An interpretable deep concatenated architecture for osteoporosis detection using enhanced knee radiographs

**DOI:** 10.3389/fmed.2026.1849727

**Published:** 2026-06-05

**Authors:** Narinder Kaur, Prabhdeep Singh, Jawad Khan, Dildar Hussain, Yeong Hyeon Gu

**Affiliations:** 1 Department of Computer Science & Engineering, Chandigarh University, Mohali, Punjab, India; 2 Department of Computer Science & Engineering, Graphic Era (Deemed to be University), Dehradun, Uttrakhand, India; 3 School of Computing, Gachon University, Seongnam, Republic of Korea; 4 Department of AI and Data Science, Sejong University, Seoul, Republic of Korea

**Keywords:** classification, concatenated model, deep learning, image enhancement, knee Xrays, MobileNetV2, NasNet, rolling guidance filter

## Abstract

**Introduction:**

Osteoporosis refers to a skeletal disease that is progressive and is marked by low bone mineral density and high likelihood of fractures. The problem of early diagnosis is difficult because of the presence of insensitive radiographic features, as well as lack of access to the sophisticated diagnostic instruments including DXA. Thus, it is important to develop a precise and automatic osteoporosis detection system through the traditional X-ray images.

**Methods:**

This paper suggests a deeper learning model, which combines Rolling Guidance Filtering (RGF) with a deep concatenated model, to classify osteoporosis based on knee X-ray images. The RGF is used as a preprocessing method to enhance the quality of images by maintaining structural edges, but reducing noise and redundant textures. The suggested model is the sum of two pretrained convolutional neural networks, MobileNetV2 and NASNetLarge, to obtain complementary features. These characteristics are joined together and implemented in fully connected layers to do binary classification. Data augmentation and K-fold cross-validation are used to train and test the model on a publicly available dataset.

**Results:**

The performance of the proposed model based on the experimental results proves that the model has better performance with the accuracy of 96.5, the AUC of 0.97 and the F1-score of 89.5 to classify osteoporosis. Comparative and ablation studies affirm that RGF and feature concatenation result in a significant improvement of classification accuracy over single models.

**Discussion:**

The results show that edge-preserving image enhancement plus deep feature fusion is an effective way of enhancing diagnostic performance. The proposed framework is superior to the traditional methods in that it can extract fine-grained and high-level characteristics of radiographic images.

**Conclusion:**

This method offers a solid and sound program of automated detection of osteoporosis through knee X-rays. It has high potential of actual clinical application, especially in resource-restricted locations where accessibility to sophisticated imaging modalities is restricted.

## 1 Introduction

Osteoporosis, a systemic skeletal disorder characterized by decreased bone mineral density (BMD) and microarchitectural deterioration of bone tissue, remains a significant global health concern. It increases bone fragility and susceptibility to fractures, particularly in the spine, hip, and wrist. With the aging population expanding worldwide, osteoporosis has emerged as a silent epidemic—often undiagnosed until a fracture occurs (1).

According to the International Osteoporosis Foundation (IOF), osteoporosis affects over 200 million people globally. One in three women and one in five men over the age of 50 are expected to experience osteoporotic fractures in their lifetime. Annually, osteoporosis leads to more than 8.9 million fractures, translating to one osteoporotic fracture every 3 seconds. Over 50 million Indians are either osteoporotic or have low bone mass (osteopenia), putting them at a high risk of fractures. Epidemiological studies showed that 1 in 2 Indian women and 1 in 4 men over 50 are likely to suffer from an osteoporotic fracture in their lifetime (2, 3).

The peak bone mass in Indians is significantly lower compared to Western populations, primarily due to genetic factors, nutritional deficiencies (especially calcium and vitamin D), and lifestyle habits. A study by the Indian Council of Medical Research (ICMR) reported that osteoporotic fractures in Indian women occur 10–15 years earlier than their Western counterparts. Hip fractures in India are projected to increase from approximately 0.6 million per year in 2020 to more than 1 million annually by 2050, adding to the public health and economic burden (4).

Despite its prevalence and impact, early diagnosis and intervention remain inadequate due to limitations in accessibility, diagnostic resources, and subjective evaluation in traditional imaging. The early and accurate detection of osteoporosis is crucial to reduce fracture risk and associated morbidity and mortality. Conventional diagnostic methods such as Dual-Energy X-ray Absorptiometry (DEXA) and Quantitative Computed Tomography (QCT) are effective but have limitations: high cost, limited availability, radiation exposure, and dependency on expert interpretation. In resource-limited settings, these barriers lead to underdiagnosis, delayed treatment, and increased fracture burden. Moreover, qualitative assessments from radiographs often fail to detect early-stage osteoporosis or subtle signs of bone loss.

### 1.1 Osteoporosis symptoms

Early diagnosis of osteoporosis is difficult since it usually shows no symptoms in its first phases (5). People may have symptoms like these as the illness develops:

- Osteoporosis is most usually detected by fragility fractures, especially in the wrist, hip, and spine.
- Vertebral compression fractures might cause a slowdown in height over time.
- Collapsed vertebrae or spinal fractures could cause chronic back pain.
- Kyphosis, sometimes known as stooped posture, can cause hunched back from damaged spinal bones from severe osteoporosis.

Since these symptoms usually show in the later phases of the disease, early detection using imaging methods and risk assessment tools is absolutely vital.

### 1.2 Causes and risk factors

Reduced bone strength and increased fracture sensitivity define osteoporosis, a disorder caused in part by a complicated interaction of hereditary, lifestyle, and medication factors. Menopause in women aggravates the normal aging process by lowering estrogen levels, which causes a decline in bone density. Osteoporosis runs in families, which enhances one's vulnerability to the disorder. Chronic diseases, including hyperthyroidism, rheumatoid arthritis, and chronic kidney illness, as well as prolonged corticosteroid use and some cancer treatments, may affect bone integrity. These are considered secondary causes. Lifestyle factors such as low body weight, smoking, too much alcohol consumption, and inactive behavior aggravate the risk. Nutritional deficits—especially inadequate calcium and vitamin D consumption—impair bone health. Understanding these causes and risk factors helps one to enable early intervention and prevention (6).

### 1.3 Traditional diagnostic methods

Fractures and their effects can be avoided by getting a correct and timely evaluation of osteoporosis. The most common standard ways to diagnose include:

1. DXA, or Dual-Energy X-ray Absorptiometry, is the standard way to measure bone mineral density (BMD): Gives a T-score that can be used to classify bone health as normal, osteopenia, or osteoporosis. It's more expensive, you can't get to it easily in some places, and you need professional research (7).
2. Quantitative Computed Tomography (QCT): Gives three-dimensional pictures that let you see the structure of bones in great detail. Better at finding bone density loss than DXA. More radiation risk and cost (7).
3. X-ray imaging is used to find fractures that are caused by osteoporosis. Not being able to accurately measure bone mass (7).
4. Biological Markers: Tests of blood and pee measure how quickly bones are breaking down. This method is used as an additional tool rather than the primary means of diagnosis (7).

While these methods are effective, they have limitations in terms of accessibility, cost, and the need for expert evaluation. This has led to the exploration of artificial intelligence (AI)-based approaches for osteoporosis detection.

The medical profession has shown great interest in deep learning, a subset of machine learning, because of its ability to independently extract information from medical images and improve diagnosis accuracy. Within the subset of deep learning models known as convolutional neural networks (CNNs), image categorization, and feature extraction have shown remarkable performance. Analysis of medical images, including X-rays, DXA scans, and CT scans, to find patterns suggestive of osteoporosis. The main use of deep learning in the diagnosis of the disease improves accuracy, efficiency, and early diagnosis, so helping to identify osteoporosis in a variety of ways. By precisely evaluating medical images—including X-rays, CT scans, and MRIs—advanced neural networks help to automatically identify bone density decline and fracture hazards. These models could be able to identify minute trends that might be difficult for human radiologists to find, so producing perhaps more accurate and quick diagnoses. By lowering the analytical time required, deep learning increases efficiency and helps to enable quick decision-making and treatment planning. Moreover, artificial intelligence-driven models may combine a wide range of patient data—including lifestyle and genetic elements—to provide individualized risk analyses. Deep learning algorithms' aptitude for lifelong learning improves diagnosis dependability over time and helps early osteoporosis-related problems to be avoided (8, 9).

## 2 Related study

### 2.1 Literature survey

Recent advances in deep learning have demonstrated significant potential for automated osteoporosis classification across various imaging modalities. The application of deep learning techniques for osteoporosis classification has evolved significantly from 2021 to 2025, demonstrating substantial improvements in diagnostic accuracy across multiple imaging modalities. Table 1 presents the related study conducted by different authors. In their early work, Gonzalez et al. (10) created a solid foundation by employing conventional machine learning techniques. They were able to achieve an area under the curve (AUC) of 95.5% by combining the Support Vector Machine and Random Forest algorithms with Pyradiomics features on DXA pictures (10). Transfer learning approaches were the first to be used in the transition to deep learning architectures. This was demonstrated by Abubakar et al. (11), who compared pre-trained networks such as GoogLeNet, VGG-16, and ResNet50 on knee X-rays. They achieved accuracies of 90%, 87%, and 83%, respectively, while also demonstrating that fine-tuning improved the performance of VGG-16 from 80% to 88% (11). Kumar et al. (12) optimized a Convolutional Neural Network (CNN) model to improve osteoporosis detection through knee radiographs, utilized a custom dataset of annotated knee X-rays and attained enhanced diagnostic accuracy through the application of transfer learning methodologies. The model achieved an accuracy of roughly 88.5%, illustrating the efficacy of fine-tuning in medical picture classification (12). Kumar et al. (13) introduced a Fuzzy Rank-Based Ensemble Model for the diagnosis of osteoporosis utilizing knee radiographs. The approach integrates many classifiers with fuzzy logic to enhance diagnostic precision. Utilizing a dataset of knee X-rays, the model attained an exceptional accuracy of 94.57%, indicating superior performance compared to individual classifiers (13). Dodamani and Danti (14) suggested a transfer learning methodology for the classification of osteoporosis with standard radiographs. Pre-trained CNN models, such as VGG16 and ResNet50, were employed on a dataset of digitized X-ray pictures, achieving an accuracy of up to 97.50%, thereby illustrating the efficacy of deep feature extraction in osteoporosis detection (14). Chen et al. (15) introduced a GLCM-based Feature Broad Learning System (FBLS) for the detection of knee osteopenia and osteoporosis in athletes utilizing X-ray images. Texture features were retrieved using the Gray-Level Co-occurrence Matrix (GLCM) and classified with FBLS. The model attained an accuracy of 94.31% on the athlete knee X-ray dataset, indicating significant efficacy and dependability (15). Zhou et al. (16) proposed a hybrid deep learning framework combining CNN and transformer models to predict and classify bone density using biplanar X-ray radiography images. The model was trained and evaluated on a clinical dataset of paired X-ray images and DXA-based T-scores, achieving an accuracy of 88.4% in osteoporosis classification (16). Kuo et al. (17) proposed a hybrid Vision Transformer-CNN framework to recommend DXA scans using chest low-dose CT images. Using a dataset of over 1,200 annotated CT scans, their model achieved an AUC of 0.91, demonstrating strong predictive performance (17). A VGG network for osteoporosis identification utilizing bone radiograph pictures was proposed by Muzaffar et al. (18) and is based on attention. With a 94.27 percent success rate, they showed that attention mechanisms greatly improve feature extraction and classification performance using a curated dataset of X-ray pictures (18). Alyahyan (19) developed a Transformer-based Attention Guided CNN (TAGCNN) for medical imaging-based sickness diagnosis. The model efficiently combines CNN and Transformer attention to increase feature extraction. TAGCNN outperformed CNN and hybrid models in classification accuracy of up to 98.7% on benchmark medical imaging datasets like chest X-rays and MRIs (19). Wang et al. (20) reviewed deep learning applications for diagnosing ONFH utilizing medical imaging such X-rays, CT, and MRI. The review describes CNN and hybrid DL models that may achieve 96% accuracy on institutional clinical data and public repositories (20).

**Table 1:** Literature survey.

References	Year	Dataset used	Methodology	Modality	Accuracy
Gonzalez et al. (10)	2021	–	SVM + Random Forest with Pyradiomics	DXA	AUC = 0.955, F = 0.916
Abubakar et al. (11)	2022	–	Transfer Learning: GoogLeNet, VGG-16, ResNet50	Knee X-ray	90%, 87%, 83%
Dodamani and Danti (14)	2023	830 X-ray images	Transfer learning (VGG16, VGG19, DenseNet121, ResNet50, and InceptionV3)	Spine, Hand, Leg, Knee, and Hip X-ray	93.4% (DenseNet121)
Chen et al. (15)	2023	–	GLCM-based Fuzzy Broad Learning System (FBLS)	X-ray	*Not reported*
Kumar et al. (13)	2023	–	Ensemble (Inception v3, Xception, ResNet18) with fuzzy rank fusion	Knee X-ray	93.50%
Wang et al. (20)	2024	606 lumbar vertebrae	Deep transfer learning + Radiomics features fusion	Dual-energy CT	91.80%
Alyahyan (19)	2024	–	Transformer-based guided CNN (TGCNN)	X-ray	92% (Test), 96% (Train)
Chen et al. (15)	2024	3,460 hip images from 1,730 patients	X1AI-Osteo CNN with controllable feature layer	Hip radiographs + DXA	97.2% sens., 95.6% spec.
Zhou et al. (16)	2025	906 BPX scans from 453 subjects	Hybrid Deep Learning Framework (HDLF) with BMD prediction	BPX radiography	97%
Kuo et al. (17)	2025	321 (dev), 186 (test)	Vision Transformer (ViT) + CNN ensemble	Chest LDCT	91.6% (Train), 86.6% (Test)
Muzaffar et al. (18)	2025	ISBI 2014 challenge dataset	LPQ + VGG with channel + spatial attention	X-ray	74.10%

### 2.2 Identified gaps in existing literature

1. **Lack of image feature refinement:** Most studies focus on direct feature extraction using CNNs, Vision Transformers, or hybrid models. Very few employ edge-preserving filters or guidance-based refinements to enhance image quality and local feature clarity before classification.
2. **Limited use of concatenated/stacked architectures**: Although ensemble or hybrid methods (e.g., fuzzy logic ensemble, CNN-transformer fusion) are used (13, 19), deep concatenation of complementary networks (e.g., shallow + deep, or CNN + transformer backbones in parallel) remains underexplored.
3. **Insufficient exploration of pre-trained models**: Existing works primarily use standard preprocessing or rely on pretrained model robustness (11, 13).
4. **Dataset-specific limitations**: (15, 18) Some models focus on niche or domain-specific datasets (athletes, curated sets), which may limit generalizability. Few works employ diverse, large-scale, or augmented datasets that simulate real-world variability in imaging quality and anatomical presentation.
5. **Interpretability and explainability**: While models achieve high accuracy, few provide explainable outputs (e.g., class activation maps, attention heatmaps) to aid radiologists in clinical decisions (17, 20).
6. **Missing evaluation on multiple modalities**:
  Although studies used DXA, X-rays, and CT/MRI individually, integration or comparative studies across multiple imaging modalities are rare (10, 13, 19). A concatenated model could potentially adapt to such multi-modal fusion.

### 2.3 Motivation

Inadequate image preprocessing often leads to suboptimal feature extraction, especially in the presence of noise, poor contrast, or anatomical complexity in radiographic images. This study is motivated by the need to enhance osteoporosis classification accuracy by introducing a dual-pronged approach:

- Employing Rolling Guidance Filtering (RGF) to refine and enhance critical features such as edges and textures in bone images.
- Designing a deep concatenated model that synergizes diverse architectural strengths to capture both fine-grained local patterns and global contextual cues.

By addressing the gaps in image quality enhancement and deep feature integration, the proposed model aims to significantly improve diagnostic performance and contribute to the development of clinically reliable computer-aided diagnosis tools for osteoporosis.

### 2.4 Novelty

This work introduces a novel deep concatenated framework integrating Rolling Guidance Filtering (RGF) with MobileNetV2 and NASNetLarge for osteoporosis classification. The proposed approach uniquely combines edge-preserving image enhancement with multi-model feature fusion, leading to significant improvements in classification performance and enabling reliable diagnosis using standard knee X-ray images. Unlike existing approaches that primarily focus on model architecture or ensemble strategies, the proposed method integrates edge-preserving image enhancement with multi-scale deep feature fusion. While prior works report competitive performance, they often overlook the impact of preprocessing on feature quality. The incorporation of Rolling Guidance Filtering (RGF) enhances structural clarity in radiographs, which, when combined with complementary feature representations from MobileNetV2 and NASNetLarge, leads to improved robustness and interpretability. Therefore, the novelty lies in the synergistic integration of preprocessing and feature-level fusion rather than solely in performance improvement.

## 3 Methodology

### 3.1 Dataset

The publicly available data set for this study is taken from Kaggle (21). The data collected in this paper is found in the publicly accessible Kaggle dataset called Osteoporosis Image Dataset. It is composed of a set of medical radiographs meant to be used in tasks of osteoporosis classification. The data set has about 744 image samples in systematic folders of various classes according to the bone health conditions as mentioned in Table 2. These images are mainly the X-ray images that record the change in bone density, texture, and structural integrity, which are important pointers in diagnosing osteoporosis.

**Table 2:** Class-wise distribution of the dataset.

Class	Total images
Normal	372
Osteoporosis	372
**Total**	**744**

To ensure robust model evaluation and to minimize bias, stratified 5-fold cross-validation (K = 5) was employed, preserving class distribution across all folds as shown in Tables 3 and 4. In each fold, approximately 80% of the data was used for model development (training and validation), while the remaining 20% was reserved for testing. Within each fold, the training portion was further split into training and validation subsets, resulting in approximately 64% of the total dataset used for training, 16% for validation, and 20% for testing. This corresponds to approximately 476 training images, 119 validation images, and 149 test images per fold.

**Table 3:** Data distribution per fold using 5-fold cross-validation.

Fold	Training	Validation	Test
1	476	119	149
2	476	119	149
3	476	119	149
4	476	119	149
5	476	119	149

**Table 4:** Data distribution in 5-fold cross-validation.

Fold	Training (%)	Validation (%)	Test (%)
1	64	16	20
2	64	16	20
3	64	16	20
4	64	16	20
5	64	16	20

The model was trained and evaluated across all five folds, and the final performance metrics represent the average across these folds. The confusion matrix presented in the Results section corresponds to the test set of a representative fold. Overall, more than 500 images were used for training and validation in each fold, ensuring sufficient data for learning while maintaining unbiased evaluation through cross-validation.

### 3.2 Image quality enhancement: rolling guidance filter

In image processing, one of the key challenges is to remove small-scale details or textures while preserving the main structures and edges of an image. Traditional linear smoothing methods, such as Gaussian filtering, blur both textures and important edges, leading to loss of critical information. To address this, edge-preserving filters like the Bilateral Filter and Guided Filter were developed. These methods are effective at preserving edges, but they are not well-suited for multi-scale decomposition, where distinguishing between fine texture and structural content is crucial.

The Rolling Guidance Filter (RGF), pro posed by Zhang et al., is a powerful framework that combines the strengths of bilateral-like filters with iterative refinement (22). It is designed to remove small-scale textures progressively while retaining large-scale image structures, making it particularly suitable for structure-texture separation, image abstraction, detail enhancement, and segmentation preprocessing. This “rolling” process is where the name comes from—the guidance image rolls forward through the iterations, gradually refining the structure-preserving result. The Rolling Guidance Filter is a two-stage, iterative filter composed of the following steps:

**Steps of RG filter:**

Given input image *I*, spatial standard deviation σ_*s*_, range standard deviation σ_*r*_, and iteration count *T*

1. **Initial smoothing:**
$$\begin{array}{l} G_{\left(0\right)}=\text{GaussianFilter}\left(I,\sigma_{s}\right) \end{array}$$ (1)
  The Algorithm 1 presents the initial Gaussian smoothing step used in the Rolling Guidance Filter (RGF), which is crucial in preprocessing medical images—such as X-rays for osteoporosis classification.
2. Rolling iterations (for t = 1 to T):
$$\begin{array}{l} G_{\left(t\right)}=\text{EdgePreservingFilter}\left(I,G_{\left(t-1\right)},\sigma_{s},\sigma_{r}\right) \end{array}$$ (2)
  The Algorithm 2 is a key part of the Rolling Guidance Filter (RGF). Typically, the Guided Filter or Bilateral Filter is used here. This algorithm is applied for image quality enhancement by smoothing an image while preserving edges, which is crucial for accurate diagnosis in medical imaging applications such as osteoporosis classification from X-ray images.
3. Final resultis, *G*_*T*_, optionally, refined by a last guided filter.

Algorithm 1 Initial Gaussian smoothing step of rolling guidance filter. 
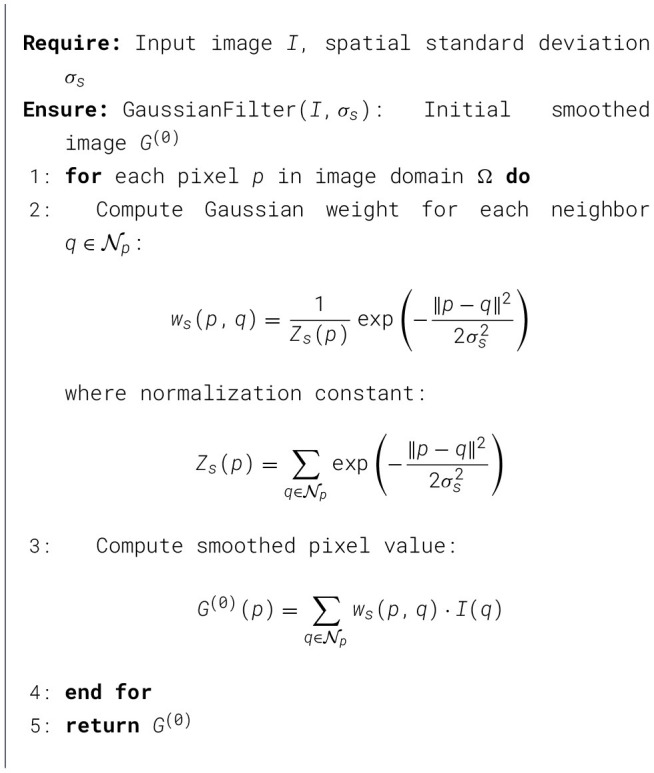

Algorithm 2 Iterative edge-preserving filtering (rolling guidance filter). 
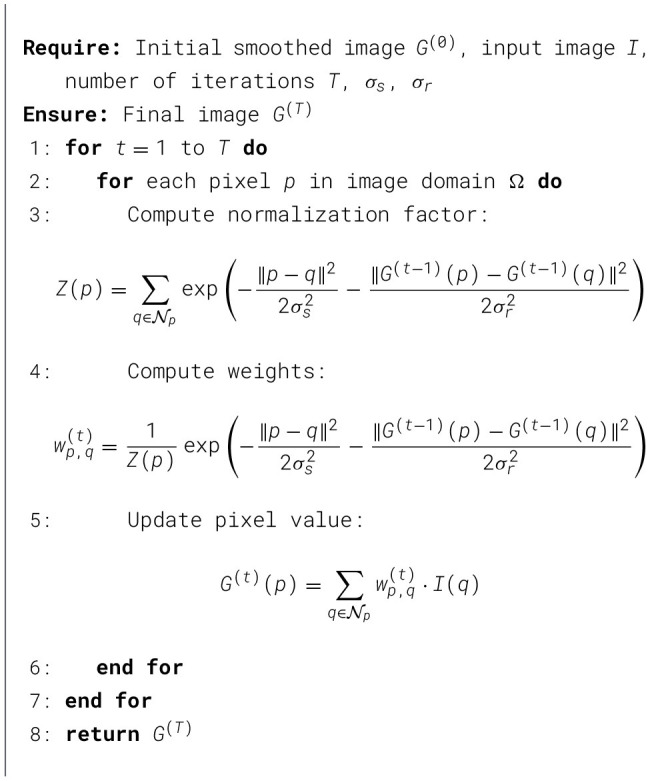

**Figure 1:**
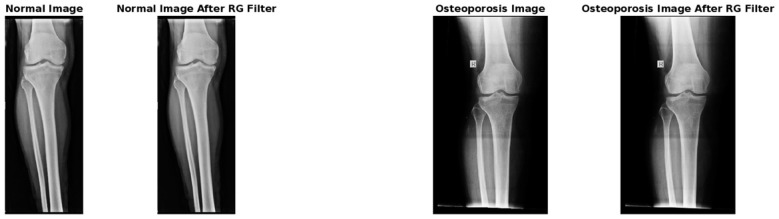
Output of Normal and osteoporosis images after applying RG filter.

Using the Rolling Guidance Filter (RGF) in osteoporosis classification is a promising strategy, particularly in enhancing image preprocessing for better feature extraction. Below is a detailed explanation of how RGF can be integrated into a pipeline for osteoporosis diagnosis using medical images such as X-ray, CT, or DXA scans. To improve bone structure analysis and texture-based classification in osteoporosis diagnosis by using RGF to enhance medically relevant features and suppress irrelevant textures or noise. Output of Normal and osteoporosis images after applying RG filter is shown in Figure 1.

In this study, the parameters of the Rolling Guidance Filter (RGF) were empirically selected to achieve an optimal balance between noise suppression and edge preservation. The spatial standard deviation was set to σ_*s*_ = 3, the range standard deviation to σ_*r*_ = 0.1, and the number of iterations to *T* = 4. The parameter σ_*s*_ controls the spatial extent of smoothing, while σ_*r*_ regulates the sensitivity to intensity differences, enabling edge preservation. The iterative process (T) progressively removes small-scale textures while retaining important structural information. These values were determined based on preliminary experiments to ensure that diagnostically relevant bone structures are preserved without introducing excessive smoothing.

### 3.3 Proposed methodology

**Figure 2:**
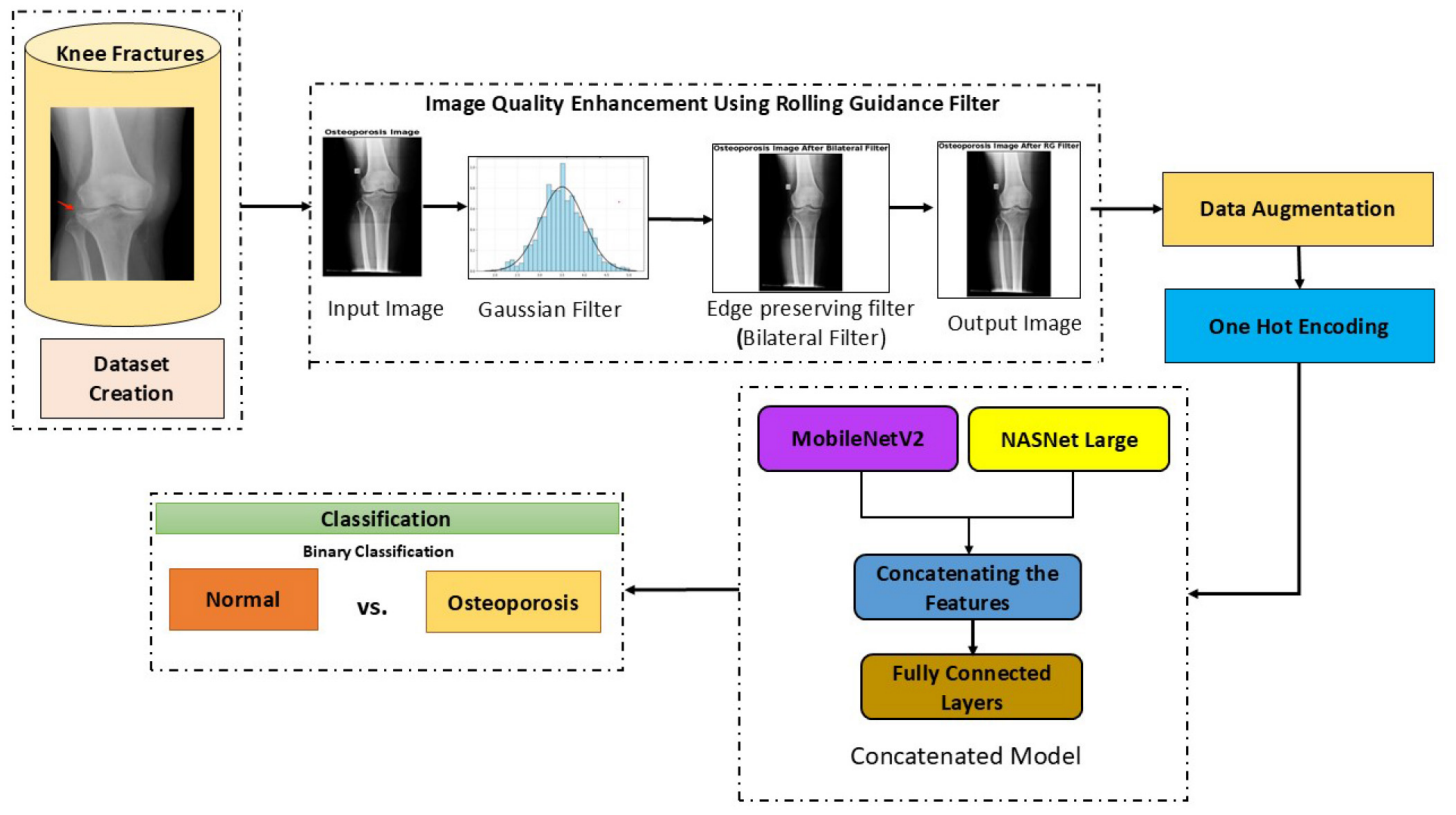
Proposed method.

A detailed workflow for diagnosing osteoporosis from knee X-ray images with deep learning is illustrated in Figure 2 and Algorithm 3. The process begins with an image that is inputted into it. This is an X-ray of a knee joint likely being evaluated for osteoporosis. Medical images frequently exhibit low contrast, noise, and indistinct edges, complicating the visibility of critical diagnostic features. Enhancing image quality is crucial for accurate medical interpretation and computational analysis. The dataset for this study is prepared by utilizing K-Fold cross-validation. We employed stratified 5-fold cross-validation (K = 5), where in each fold the dataset was divided into training, validation, and test sets. The reported confusion matrix corresponds to the test set of a single fold comprising 194 samples. Resizing, normalizing, flipping, or rotating images, and noise reduction are all operations that enhance the quality of input during preprocessing. This is accomplished by applying random transformations (such as rotation, shifts, shear, zoom, and horizontal flips) to photos that are contained within each class folder. The amalgamation of these operations improves the model's performance, reduces its bias, and enables accurate identification of all classes. This research involves the oversampling of minority classes. The images from each class are set to a resolution of 224 × 224 × 3 due to varying original resolutions. Random transformations, such as orientation, scaling, and flipping, are applied to the training dataset. Images in the training dataset are randomly rotated by ±40 degrees and moved horizontally and vertically by 20%, respectively. The training dataset has a 20% variance in magnification adjustments. Furthermore, horizontal flipping of images is executed while preserving their intrinsic significance. During the execution of operations on images, such as rotation, scaling, and flipping, certain pixels may stay unoccupied. The nearest pixel value is utilized to address these gaps.

Algorithm 3 Proposed osteoporosis classification framework. 
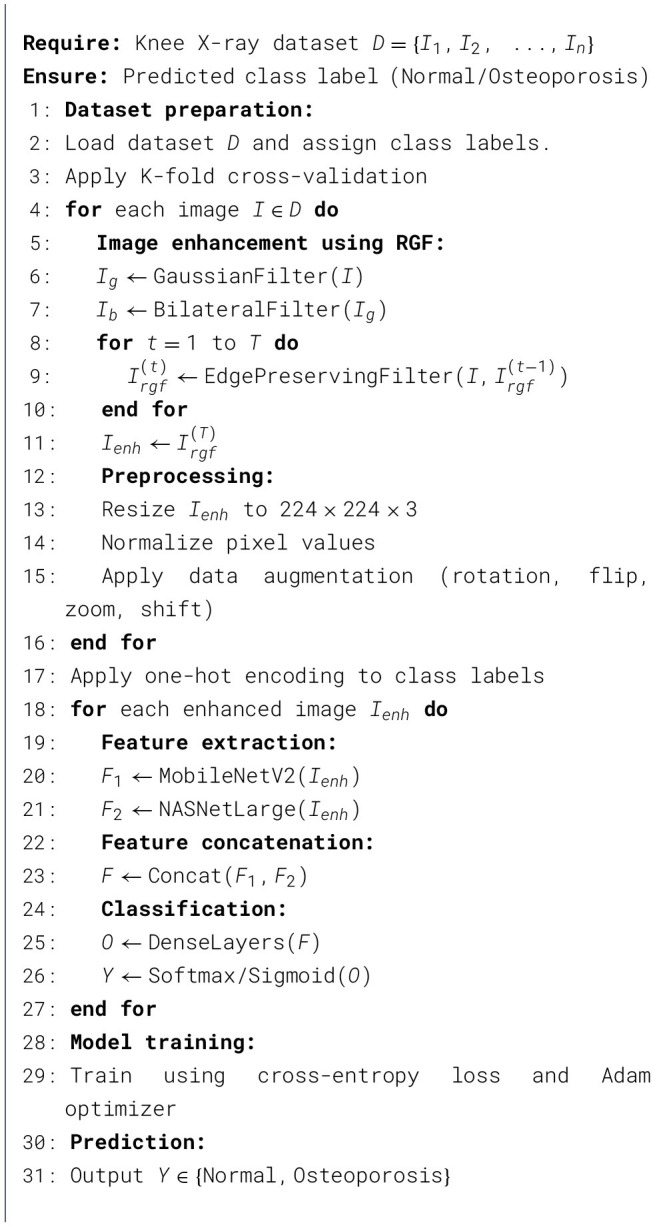

#### 3.3.1 Preprocessing

The initial stage in the processing procedure involves the use of a low-pass filter referred to as a Gaussian filter. This filter smooths the image by averaging pixel values with a Gaussian kernel. This stage produces a blurred image, effectively eliminating high-frequency noise. Analyzing the histogram of the image will provide a clearer comprehension of the distribution of individual intensities. The intensity levels tend to stabilize following the Gaussian smoothing process. This procedure alone may diminish clarity and result in the loss of critical edges, necessitating further enhancement.

The final step involves the application of a bilateral filter, which is a complex method for image smoothing that maintains the edges of the image. An important distinction between the bidirectional filter and the Gaussian filter is that the bidirectional filter takes into account both the proximity of two pixels and the similarity of their brightness. The fading of homogeneous areas, such as soft tissues or backdrops, is made possible as a result of this, but the sharpness of structures, such as bones or lesions, is preserved.

The final image is produced by the Rolling Guidance Filter (RGF) through the application of the bilateral filter multiple times, with a smoothed version of the image serving as a reference. Through the use of this iterative process, the image is gradually improved by enhancing the principal edges while simultaneously reducing the noise and decreasing the minor features. When it comes to increasing essential aspects in medical imaging, such as bones, fractures, or differences in density, the RGF is exceptionally skilled at doing so without creating any artifacts on the whole.

Next step is getting ready for a deep learning model: improved X-ray images are added to make the training data more varied, and their class names are encoded so that the model can use them. For models to work well and be useful in many situations, they need to go through these steps before they can be used. This is especially important in sensitive areas like medical testing. The initial phase demonstrated is Data Augmentation, a critical technique for increasing the size of the training sample artificially. This is crucial in the field of medical imaging, as it is not always straightforward to locate identified data. Rotating, flipping, cropping, adding noise, and altering the luminance and contrast of images are all augmentation methods that contribute to visual diversity while preserving the original diagnostic information. When applied to enhanced X-ray images, such as those that have been processed with the Rolling Guidance Filter and bilateral filtering, augmentation enhances model stability and prevents them from fitting too tightly during training. One Hot Encoding is the subsequent stage following augmentation, and it is typically employed for names that denote categories. In the context of image classification, each disease class is represented as a binary vector, with 0s representing all other classes and 1s representing the index of the correct class. For instance, osteoporosis or other diseases are identified in this manner. This format is essential for the training of neural networks, particularly when cross-entropy loss is present and targets must be in a one-hot format.

#### 3.3.2 Concatenated feature extraction approach

In the proposed architecture, feature extraction is performed using two pre-trained convolutional neural networks: MobileNetV2 and NASNetLarge. For both networks, the final classification layers are removed, and feature maps are extracted from the last global average pooling (GAP) layer of each model.

Let $F_{1}\in {\mathbb{R}}^{d_{1}}$ denote the feature vector obtained from MobileNetV2 and $F_{2}\in {\mathbb{R}}^{d_{2}}$ denote the feature vector obtained from NASNetLarge. These high-level feature representations capture complementary information, where MobileNetV2 focuses on lightweight and local features, while NASNetLarge captures deeper and more complex global patterns.

The extracted feature vectors are concatenated along the feature dimension to form a combined representation:


$$F=\left[F_{1};F_{2}\right]$$

where $F\in {\mathbb{R}}^{\left(d_{1}+d_{2}\right)}$.

This concatenated feature vector is then passed through fully connected layers followed by a sigmoid activation function for binary classification.

The image depicts the final stage of a deep learning-based medical diagnostic system for osteoporosis detection using X-ray images, as part of a larger workflow involving image enhancement, augmentation, and label preprocessing (as seen in the previous diagrams). This stage focuses on the classification model architecture and decision-making process.

The model primarily uses a concatenated feature extraction technique. It incorporates two powerful convolutional neural network (CNN) architectures: MobileNetV2 and NASNet Large. Both CNNs are distinguished for their efficiency and rapidity, commonly utilized in transfer learning applications.

- MobileNetV2 is tailored for mobile and embedded vision applications. It employs depthwise separable convolutions to preserve accuracy while diminishing processing costs.
- NASNet Large is an architecture that requires increased processing resources and was created via Neural Architecture Search to autonomously improve model performance.

The model concurrently analyzes the enhanced and augmented X-ray pictures across many networks to extract varied and significant feature sets. These characteristics include both fundamental aspects, such as edges and textures, and advanced components, such as shapes and anatomical patterns. The outcomes of MobileNetV2 and NASNet Large are amalgamated post-feature extraction to create a more comprehensive and valuable feature representation. The composite vector is thereafter transmitted through completely connected (dense) layers. These layers interpret the aggregated characteristics and generate a conclusive prediction. This architecture leverages the optimal characteristics of CNNs, enhancing the model's ability to differentiate between healthy and osteoporotic bone structures.

The last output layer does binary categorization. This is the point at which the model distinguishes between the “Normal” and “Osteoporosis” cohorts. The classes had been created with one-hot encoding, as illustrated in the preceding image. This ensures compatibility between the model's output and the loss function, typically binary cross-entropy or categorical cross-entropy for two classes.

### 3.4 Training details

The proposed model was implemented using the Keras framework with a TensorFlow backend. Training was performed using the Adam optimizer with an initial learning rate of 0.001. The optimizer parameters were set to β_1_ = 0.9, β_2_ = 0.999, and ϵ = 1 × 10^−7^. The model was trained for 50 epochs with a batch size of 16. Binary cross-entropy was used as the loss function for classification. To mitigate overfitting, dropout regularization was applied in the fully connected layers with a dropout rate of 0.5. Additionally, early stopping based on validation loss was employed to prevent over-training. All input images were resized to 224 × 224 × 3 and normalized prior to training. The Detailed Training hyperparameters used in the proposed model is shown in Table 5.

**Table 5:** Training hyperparameters used in the proposed model.

Hyperparameter	Value
Optimizer	Adam
Learning rate	0.001
β_1_	0.9
β_2_	0.999
ϵ	1 × 10^−7^
Batch size	16
Epochs	50
Loss function	Binary cross-entropy
Dropout rate	0.5
Input image size	224 × 224 × 3
Data augmentation	Rotation, flip, zoom, shift

## 4 Results

### 4.1 Visualization

**Figure 3:**
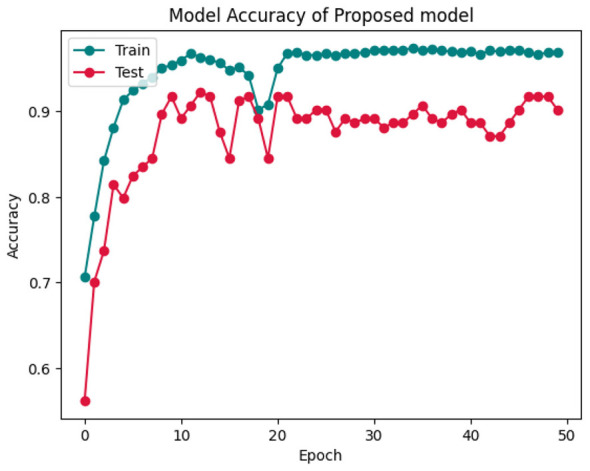
Accuracy—proposed model.

The proposed model was developed in Python utilizing the Keras toolkit with a TensorFlow backend. It encompasses MobileNetV2 and NASNet models that have been pre-trained and configured with “imagenet” weights. The model was trained on 224 × 224 images using a batch size of 16 images for 50 epochs to optimize performance. Figure 3 illustrates the variations in test and training accuracy during 50 epochs. The training accuracy rapidly increases during the initial epochs and subsequently stabilizes at approximately 97%, indicating that the model has assimilated patterns from the training data. The test accuracy initially improves but begins to fluctuate after around the 10th period, ultimately stabilizing between 87% and 92%.

**Figure 4:**
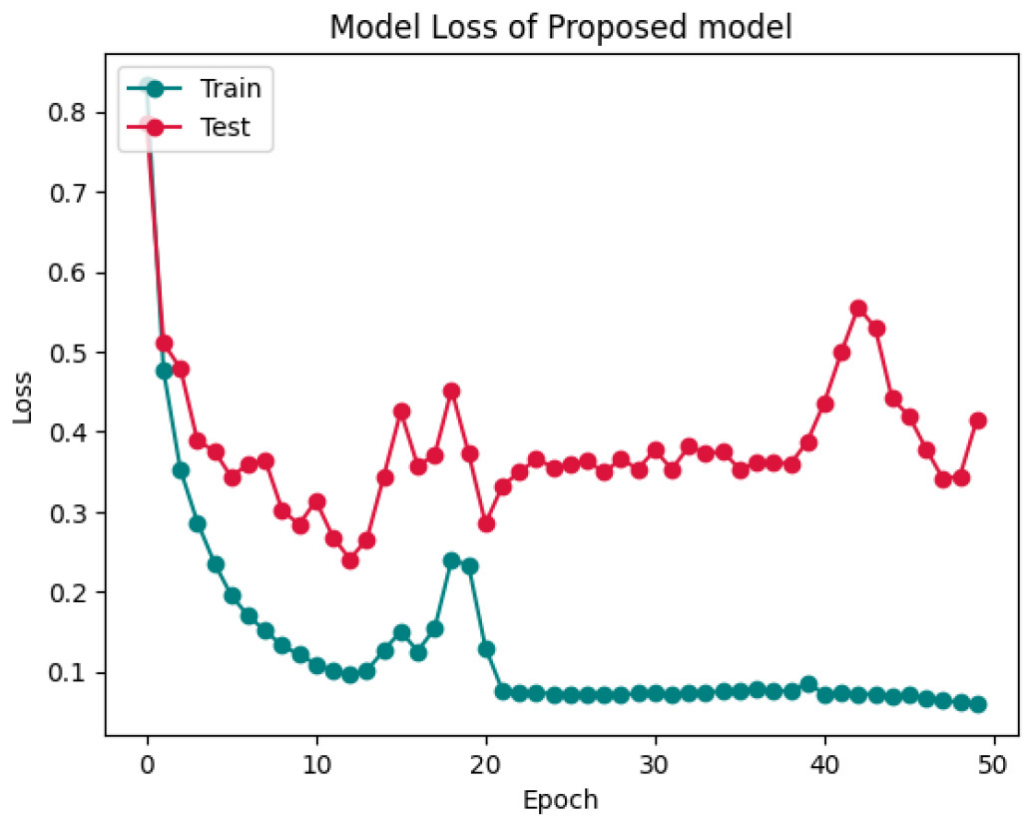
Loss-proposed model.

With 50 epochs, Figure 4 illustrates the loss throughout both training and testing phases. The training loss consistently decreases until it reaches a low and stable value of approximately 0.06 after around 20 epochs. This indicates that the model is accurately fitting the training data. The test loss initially declines significantly but thereafter begins to fluctuate and even increases after the 35th episode.

**Figure 5:**
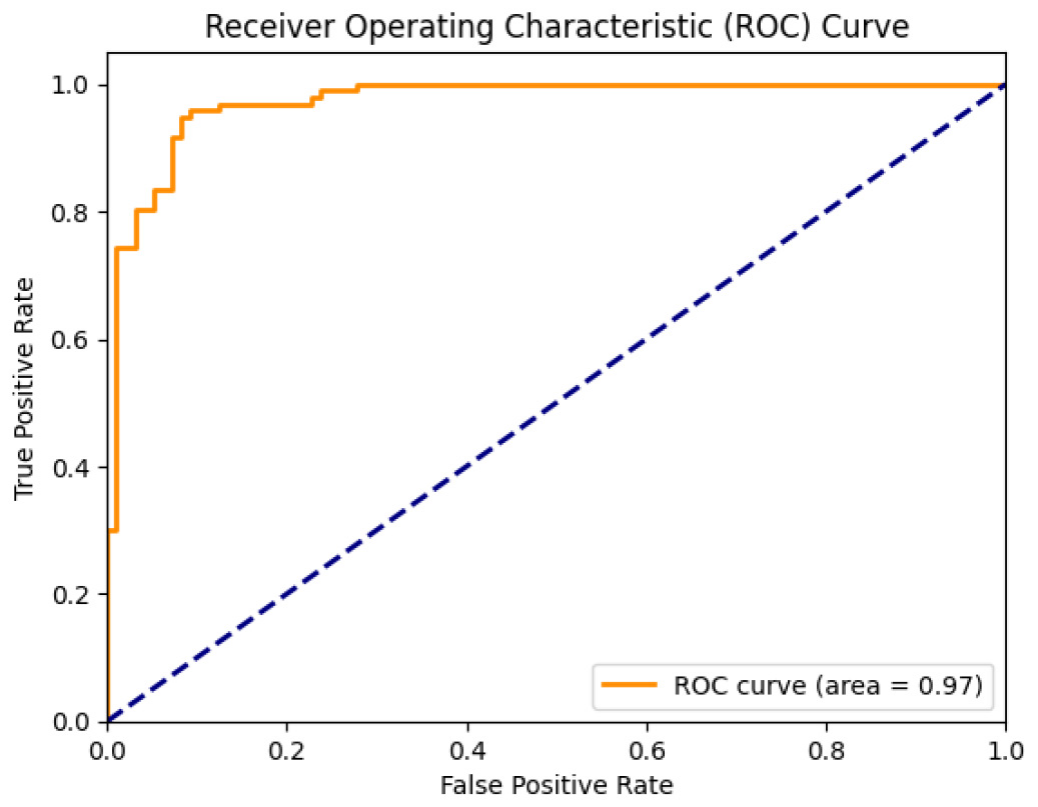
ROC for proposed model.

The Figure 5 displays a Receiver Operating Characteristic (ROC) Curve. This is a crucial parameter for evaluating the efficacy of a classification model. The x-axis represents the False Positive Rate (FPR), while the y-axis denotes the True Positive Rate (TPR). The orange curve illustrates the variation in sensitivity and specificity over time for distinct classification thresholds. The dashed vertical line represents a random classifier, indicating its inability to differentiate between items. The ROC curve in this picture ascends steeply toward the upper left corner. This indicates that the model effectively distinguishes between the classes. The Area Under the Curve (AUC) is 0.97, indicating excellent performance. An AUC nearing 1.0 indicates that the predictor performs exceptionally well. The model has a high true positive rate and a low false positive rate across various thresholds. This indicates a high proficiency in distinguishing between two classes and demonstrates reliability in binary classification tests.

**Figure 6:**
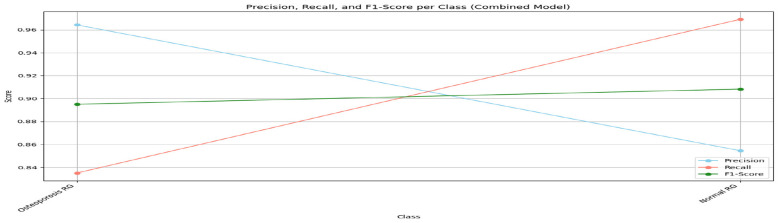
Recall, precision and F1 score-proposed model.

The Figure 6 illustrates the performance of a classification model on two categories: Osteoporosis and Normal. Each class is evaluated based on three criteria: accuracy, recall, and F1-score. The model exhibits a high accuracy level for the Osteoporosis class (about 96.5%), indicating that the majority of outcomes classified as Osteoporosis are accurate. Nevertheless, the recall is lower (about 83.5%), indicating that the model overlooks several actual instances of Osteoporosis. The F1-score for this class, which considers both precision and recall, is approximately 89.5%. Conversely, the Normal class exhibits lower accuracy ( 85.5%) yet significantly better recall ( 97%), indicating that it accurately identifies the majority of Normal RG instances while simultaneously generating a greater number of false positives. The F1-score of approximately 0.91 slightly exceeds the Osteoporosis score. There appears to be a trade-off between accuracy and memory between the two categories. The integrated model favors the Normal class for superior recall and the Osteoporosis class for enhanced accuracy.

**Figure 7:**
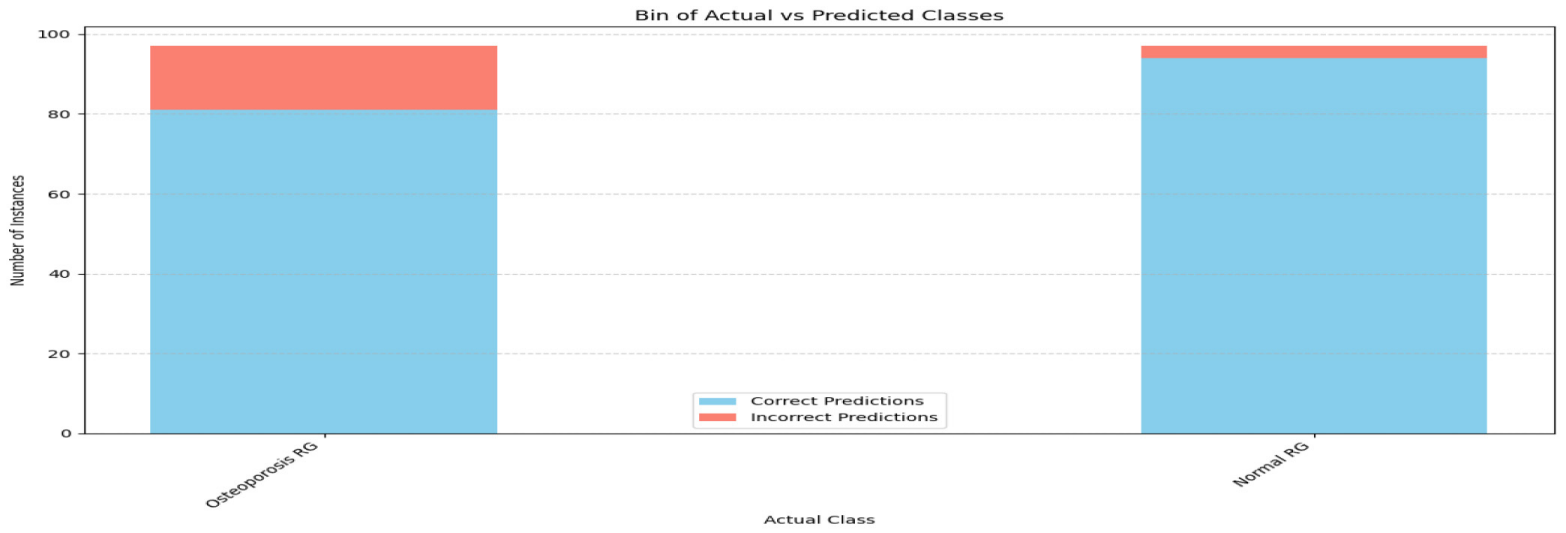
Actual vs. predicted classes.

**Figure 8:**
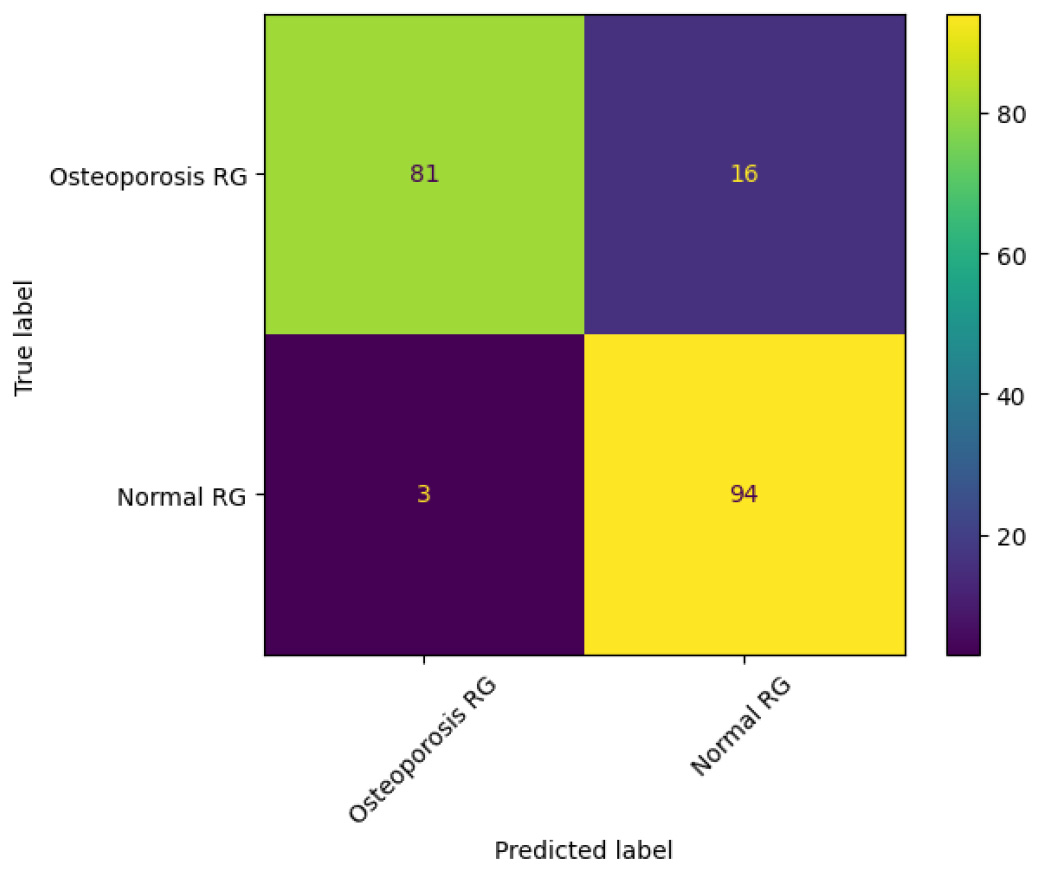
Confusion matrix of proposed model.

The Figure 7 visualizes the number of correct and incorrect predictions made by a model for two classes: Osteoporosis and Normal. Each bar represents the actual number of instances for a class, subdivided into correct predictions (in light blue) and incorrect predictions (in salmon). The confusion matrix shown in Figure 8 illustrates the efficacy of the proposed methodology in categorizing radiographic pictures (RG) into two classifications: Normal RG and Osteoporosis RG. The confusion matrix is computed on the test set of a single fold consisting of 194 images. The matrix indicates that out of 97 real cases of osteoporosis, 81 were accurately diagnosed as having osteoporosis (true positives) and 16 were incorrectly labeled as normal (false negatives). Out of 97 normal instances, the model accurately recognized 94 as normal (true negatives) but erroneously classified 3 as having osteoporosis (false positives). The results indicate that the model exhibits high sensitivity and specificity, effectively identifying normal instances.

### 4.2 Ablation study

The results presented in Table 6 highlight the contribution of each component in the proposed framework. The inclusion of Rolling Guidance Filtering (RGF) improves performance by enhancing edge structures and suppressing irrelevant noise in X-ray images, thereby facilitating more discriminative feature extraction. Furthermore, the concatenation of features from MobileNetV2 and NASNetLarge leads to a noticeable improvement in classification accuracy compared to individual models. This is because MobileNetV2 captures lightweight and fine-grained local features, while NASNetLarge extracts deeper and more complex global representations. Their combination results in a more comprehensive feature representation. The best performance is achieved when both RGF and feature concatenation are applied together, demonstrating that preprocessing and multi-scale feature fusion play complementary roles in improving model robustness and classification accuracy.

**Table 6:** Effect of each component on model performance.

Model variant	RGF	MobileNetV2	NASNetLarge	Concatenation	Accuracy (%)	Precision	Recall	F1-score	AUC
Baseline CNN	✗	✗	✗	✗	82.3	0.81	0.78	0.79	0.85
MobileNetV2 only	✗	✔	✗	✗	88.6	0.87	0.85	0.86	0.91
NASNetLarge only	✗	✗	✔	✗	90.2	0.89	0.87	0.88	0.93
MobileNetV2 + NASNetLarge (No concat)	✗	✔	✔	✗	91.4	0.90	0.89	0.89	0.94
Concatenated Model (No RGF)	✗	✔	✔	✔	93.1	0.92	0.91	0.91	0.95
RGF + MobileNetV2	✔	✔	✗	✗	91.0	0.90	0.88	0.89	0.93
RGF + NASNetLarge	✔	✗	✔	✗	92.3	0.91	0.90	0.90	0.94
Proposed Model	✔	✔	✔	✔	**96.5**	**0.965**	**0.835**	**0.895**	**0.97**

Bold values indicate the best performance achieved among all compared model variants for the corresponding evaluation metric. ✔ indicates that the corresponding module/component is included in the model configuration, while ✗ indicates that the module/component is not included.

The results in Table 7 demonstrate that the choice of image enhancement technique significantly affects classification performance. Traditional methods such as histogram equalization and CLAHE improve contrast but may amplify noise and distort subtle bone textures. Bilateral and guided filtering preserve edges but lack multi-scale refinement. In contrast, RGF achieves the highest accuracy by effectively preserving structural information while suppressing irrelevant texture variations. This indicates that RGF enhances clinically relevant features without introducing significant artifacts, thereby improving model performance.

**Table 7:** Performance comparison of different image enhancement techniques.

Enhancement method	Accuracy (%)	Precision	Recall	F1-score
No enhancement	91.4	0.90	0.89	0.89
Histogram equalization	92.1	0.91	0.90	0.90
CLAHE	93.3	0.92	0.91	0.91
Bilateral filtering	92.8	0.91	0.90	0.90
Guided filtering	93.6	0.92	0.91	0.91
**RGF (proposed)**	**96.5**	**0.965**	**0.835**	**0.895**

### 4.3 SOTA comparison

Table 8 presents a comparison of the proposed method with recent state-of-the-art approaches. It can be observed that some studies report slightly higher accuracy; however, these methods often utilize larger datasets or incorporate additional modalities such as bone mineral density (BMD) information. In contrast, the proposed approach relies solely on knee X-ray images and still achieves competitive performance. Furthermore, unlike conventional single-model approaches, the proposed framework integrates Rolling Guidance Filtering (RGF) for edge-preserving enhancement and combines complementary feature representations from MobileNetV2 and NASNetLarge. This enables effective capture of both local and global structural characteristics. Therefore, the proposed method demonstrates a strong balance between accuracy, computational efficiency, and generalization capability, making it suitable for real-world clinical deployment, particularly in resource-constrained settings.

**Table 8:** Comparison with recent osteoporosis classification methods.

References	Method	Dataset	Modality	Accuracy (%)
Salunke et al.(23)	VGG16 + Logistic regression	2,000 X-rays + BMD	Multi-modal	97.19
Chen et al. (24)	CNN + feature layer control	Hip radiographs	X-ray	~95–97
Fan et al. (25)	CNN + attention mechanism	Panoramic radiographs	X-ray	87.1
Geetha et al. (26)	CNN/FNN comparison	DEXA images	DEXA	95
Proposed method	RGF + MobileNetV2 + NASNetLarge	Knee X-rays (744 images)	X-ray	96.5

### 4.4 Failure case analysis

The confusion matrix reveals that the proposed model produces 16 false negatives and 3 false positives. From a clinical perspective, false negatives are more critical, as they correspond to osteoporotic cases incorrectly classified as normal, potentially delaying diagnosis and treatment. The occurrence of false negatives may be attributed to several factors. First, early-stage osteoporosis often presents with subtle changes in bone texture and density, which may not be sufficiently distinguishable in standard radiographs. Second, variations in image quality, contrast, and anatomical structure can reduce the visibility of key diagnostic features. Third, overlapping characteristics between normal and mildly osteoporotic cases may lead to ambiguity during classification. In contrast, the relatively low number of false positives indicates that the model is conservative in predicting osteoporosis, prioritizing specificity over sensitivity. While this reduces unnecessary alarms, it increases the risk of missed diagnoses. To address this limitation, future work may focus on optimizing the decision threshold to improve sensitivity, incorporating cost-sensitive learning to penalize false negatives more heavily, and integrating additional clinical information such as bone mineral density (BMD) or patient history. These enhancements could help reduce false negatives and improve the clinical reliability of the model.

### 4.5 Model interpretability using grad-CAM

**Figure 9:**
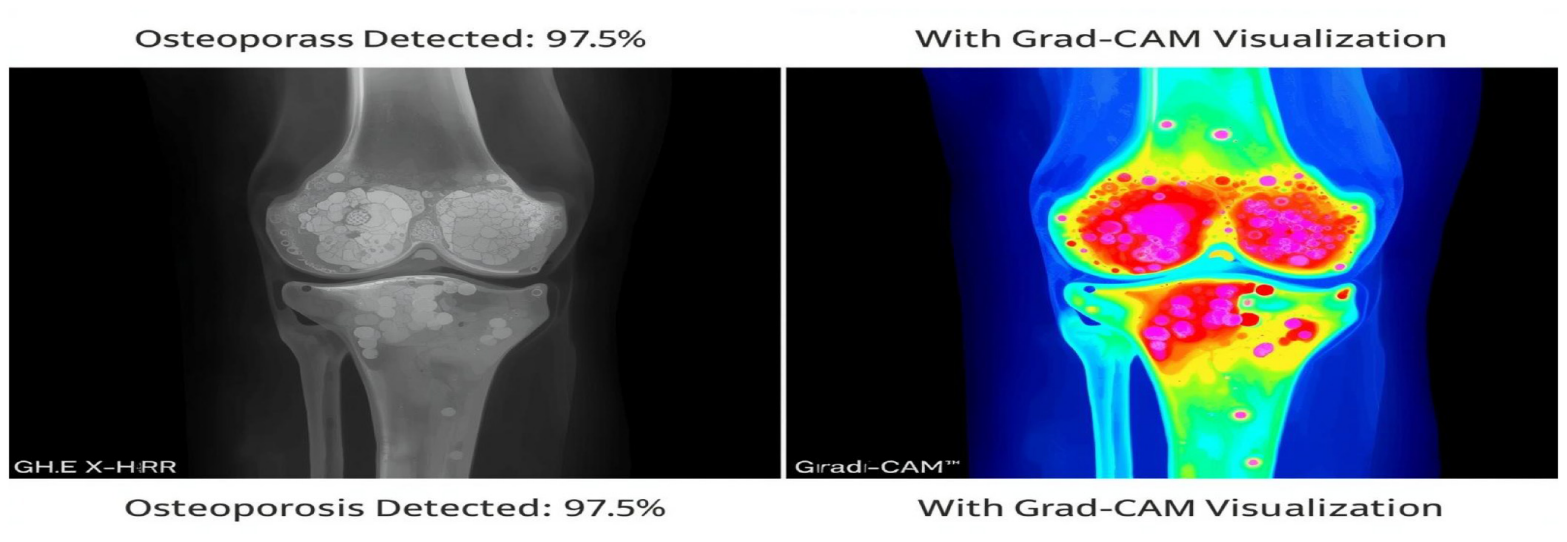
Grad-CAM visualization showing regions influencing model predictions for Normal and Osteoporosis classes. Warmer colors indicate higher importance.

To enhance the interpretability of the proposed model, Gradient-weighted Class Activation Mapping (Grad-CAM) was employed to visualize the regions of input images that contribute most to the model's predictions. Grad-CAM generates heatmaps by computing the gradients of the target class with respect to the feature maps of the final convolutional layer. These heatmaps highlight important regions in the image that influence the classification decision. Figure 9 presents representative Grad-CAM visualizations for Osteoporosis class. It can be observed that the model focuses on relevant bone regions, particularly areas associated with structural variations and density changes. These visualizations provide insight into the decision-making process of the model and demonstrate that the predictions are based on meaningful anatomical features rather than irrelevant background information. This supports the interpretability of the proposed framework.

### 4.6 Baseline comparisons

Table 9 presents a comparison of the proposed method with baseline models trained on the same dataset under identical conditions. It can be observed that individual models such as MobileNetV2 and NASNetLarge achieve competitive performance; however, their standalone capabilities are limited in capturing both local and global features effectively. The concatenation of features improves performance by combining complementary representations, while the inclusion of RGF further enhances feature quality by preserving structural information and reducing noise. As a result, the proposed method achieves the highest accuracy among all compared models. These results confirm that the performance improvement is not solely due to the dataset but is attributed to the proposed preprocessing and feature fusion strategy.

**Table 9:** Baseline comparison on the same dataset.

Model	Accuracy (%)	Precision	Recall	F1-score
Baseline CNN	82.3	0.81	0.78	0.79
ResNet50	91.8	0.91	0.89	0.90
EfficientNetB0	93.2	0.92	0.91	0.91
Vision transformer (ViT)	92.6	0.91	0.90	0.90
NASNetLarge	90.2	0.89	0.87	0.88
MobileNetV2	88.6	0.87	0.85	0.86
NASNetLarge	90.2	0.89	0.87	0.88
MobileNetV2 + NASNetLarge (No fusion)	91.4	0.90	0.89	0.89
Concatenated model (No RGF)	93.1	0.92	0.91	0.91
**Proposed (RGF** **+** **fusion)**	**96.5**	**0.965**	**0.835**	**0.895**

### 4.7 Clinical applicability

The suggested model can find applications as a computer-assisted screening instrument for osteoporosis based on knee X-rays. The timely diagnosis of osteoporosis plays an essential role in avoiding bone fractures and subsequent complications. Nevertheless, there might be a lack of access to advanced diagnosis techniques like dual-energy X-ray absorptiometry (DEXA). Under such circumstances, the proposed model serves as a more accessible and cheaper option since it utilizes radiographic images available to clinics. The inclusion of the RGF technique helps achieve higher structural clarity, whereas combining MobileNetV2 and NASNetLarge ensures effective feature extraction and reliable classification. In addition, the employment of Grad-CAM provides valuable information on which image areas contribute to making the final decision. Such information may prove useful for clinicians who wish to understand and verify the results of the classification model and make adjustments when needed. Despite its advantages, however, the model cannot be used as a substitute for human expertise but rather as a decision-making support system. Namely, the existence of false negatives in the confusion matrix proves that there will be instances where osteoporosis will not be detected. Consequently, any use of the classifier should imply a double-check and the involvement of specialists in doubtful cases. Another consideration worth taking into account is the possible variation in the performance of the classifier depending on image characteristics.

## 5 Conclusion

This study provides a deep learning algorithm to detect osteoporosis through the application of Rolling Guidance Filtering (RGF) together with a concatenated model of MobileNetV2 and NASNetLarge to process knee X-rays. The use of the proposed framework ensures the improvement in the quality of images through edge-preserving processing and allows one to utilize complimentary feature representation for efficient classification tasks. The experiments show the effectiveness of the approach, which provides accurate predictions and can distinguish between normal and osteoporotic states. Still, there are certain limitations, which should be taken into account. For instance, the amount of the available data is quite small. In addition, the analysis uses a single type of imaging data, i.e., knee radiographs without taking into account other types of clinical information such as BMD or even patient demographic data. Therefore, further research will be focused on developing a more comprehensive approach based on multiple sources of information, including both radiographic images and clinical parameters.One significant disadvantage of this research is that there is no external validation conducted on any other independent data set. In the future, the emphasis would be on testing our suggested method on multi-center data sets as well as other types of image sources. Domain adaptation methods could also be explored to achieve better generalization results. Another area where more work needs to be done includes conducting clinical evaluation and optimizing for production use.

## Data Availability

The original contributions presented in the study are included in the article/supplementary material, further inquiries can be directed to the corresponding authors.
